# Transcriptome Patterns of *BRCA1*- and *BRCA2*- Mutated Breast and Ovarian Cancers

**DOI:** 10.3390/ijms22031266

**Published:** 2021-01-28

**Authors:** Arsen Arakelyan, Ani Melkonyan, Siras Hakobyan, Uljana Boyarskih, Arman Simonyan, Lilit Nersisyan, Maria Nikoghosyan, Maxim Filipenko, Hans Binder

**Affiliations:** 1 Group of Bioinformatics, Institute of Molecular Biology National Academy of Sciences of Armenia, 0014 Yerevan, Armenia; sirashakobyan@gmail.com (S.H.); rmnsimonyan@gmail.com (A.S.); l_nersisyan@mb.sci.am (L.N.); marianikoghosyan@gmail.com (M.N.); 2 Institute of Biomedicine and Pharmacy, Russian-Armenian University, 0051 Yerevan, Armenia; 3 Laboratory of Human Genomics and Immunomics, Institute of Molecular Biology National Academy of Sciences of Armenia, 0014 Yerevan, Armenia; ani.melqonya@gmail.com; 4 Institute of Chemical Biology and Fundamental Medicine, Siberian Branch of the Russian Academy of Sciences (SB RAS), 630090 Novosibirsk, Russia; boyarskih.u@gmail.com (U.B.); max@niboch.nsc.ru (M.F.); 5 Interdisciplinary Centre for Bioinformatics, University of Leipzig, D-04107 Leipzig, Germany; binder@izbi.uni-leipzig.de

**Keywords:** *BRCA1*, *BRCA2*, somatic and germline mutations, breast cancer, ovarian cancer, transcriptome portrayal, multi-layer self-organizing maps

## Abstract

Mutations in the *BRCA1* and *BRCA2* genes are known risk factors and drivers of breast and ovarian cancers. So far, few studies have been focused on understanding the differences in transcriptome and functional landscapes associated with the disease (breast vs. ovarian cancers), gene (*BRCA1* vs. *BRCA2*), and mutation type (germline vs. somatic). In this study, we were aimed at systemic evaluation of the association of *BRCA1* and *BRCA2* germline and somatic mutations with gene expression, disease clinical features, outcome, and treatment. We performed *BRCA1/2* mutation centered RNA-seq data analysis of breast and ovarian cancers from the TCGA repository using transcriptome and phenotype “portrayal” with multi-layer self-organizing maps and functional annotation. The results revealed considerable differences in *BRCA1*- and *BRCA2*-dependent transcriptome landscapes in the studied cancers. Furthermore, our data indicated that somatic and germline mutations for both genes are characterized by deregulation of different biological functions and differential associations with phenotype characteristics and poly(ADP-ribose) polymerase (PARP)-inhibitor gene signatures. Overall, this study demonstrates considerable variation in transcriptomic landscapes of breast and ovarian cancers associated with the affected gene (*BRCA1* vs. *BRCA2*), as well as the mutation type (somatic vs. germline). These results warrant further investigations with larger groups of mutation carriers aimed at refining the understanding of molecular mechanisms of breast and ovarian cancers.

## 1. Introduction

The *BRCA1* and *BRCA2* genes play an important role in maintaining genomic integrity and tumor suppression through the mediation of DNA repair by homologous recombination and reactivation of replication [1,2]. The prevalence of germline *BRCA1* and *BRCA2* mutations is 5–15% in breast cancer (BC) [3] and 10–25% of ovarian cancer (OV) patients [4,5,6,7]. Along with the mutation carriers, the cumulative risk is around 70% and 14–44% for breast and ovarian cancers, respectively [8]. The effects of *BRCA1* and *BRCA2* on survival are also being intensively studied, although with conflicting results. In breast cancers, most of the studies have reported worse clinical prognosis for patients with *BRCA1* mutations compared with *BRCA2* mutation carriers and non-carriers [9,10,11]. Meanwhile, in other studies, no difference in survival [12], the worse outcome for *BRCA2* mutations [13], and favorable outcomes for *BRCA1* mutations were found [14]. Similarly, discrepant results were also observed in ovarian cancers. While favorable prognosis was mostly observed for carriers of *BRCA1* and *BRCA2* mutations [6,15,16], some studies attribute better survival exclusively to *BRCA2* [17], and others report no difference [18]. The mechanisms driving the differential association between *BRCA1* and *BRCA2* mutations with progression and survival in breast and ovarian cancers are not completely understood. On a molecular level, few studies were aimed at addressing this issue. The study by Jazaeri et al. (2002) has shown that there are considerable differences in transcriptome profiles in ovarian cancer patients bearing mutations in *BRCA1* or *BRCA2*, which suggests activation of different mechanisms of the disease [19]. In much the same way, few studies found differences in gene expression profiles in *BRCA1*-linked breast cancer patients compared with non-carriers [20,21]. At the same time, little is known about transcriptome associated molecular effects of somatic and germline mutations in either of the BRCA genes. In this study, we aimed at systemic evaluation of the relationships between *BRCA1* and *BRCA2* germline and somatic mutations, associated gene expression, disease clinical features, outcome, and treatment.

## 2. Results

### 2.1. Self-organizing Maps (SOM) Portrayal of Transcriptome Landscape in BC and OV

RNA-seq data from 338 ovarian cancer (TCGA-OV) and 694 breast cancer (TCGA-BRCA) samples were analyzed using two-layer self-organizing maps (Table 1). Only primary tumor samples with mutually exclusive mutations were included in the analysis.

**Figure 1:**
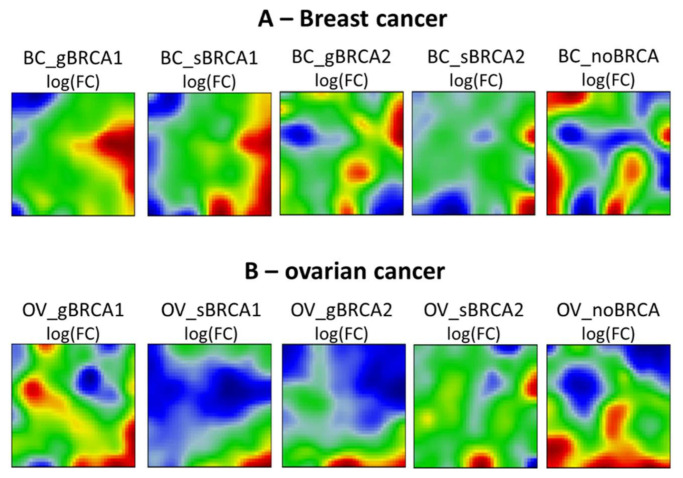
Group-specific mean expression portraits of breast (**A**) and ovarian (**B**) cancers (see Table 1). Spot-like red and blue areas indicate clusters of genes that are concertedly up- or down-regulated in each of the group portraits. Notably, genes are position-invariant in all portraits, meaning that all portraits can be compared each with another.

Multilayer SOM provided group-specific mean expression portraits averaged over all single-sample portraits per condition studied (Figure 1). These red and blue colored spot-like areas correspond to co-expressed gene clusters with correlated profiles that demonstrate high- or low expression in the different sample groups, respectively.

### 2.2. Differential Gene Expression Patterns between the BC and OV Cancer Groups

**Figure 2:**
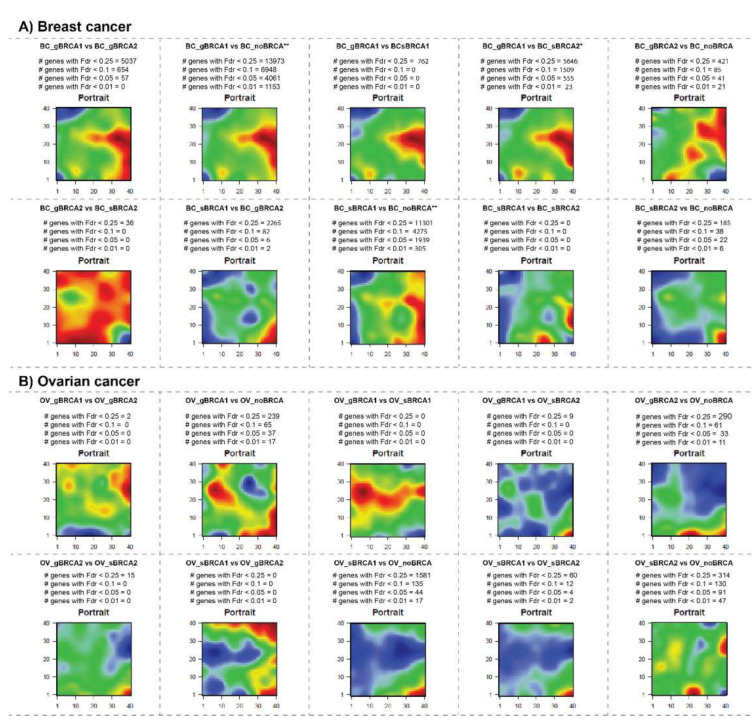
Pairwise differential expression portraits in the breast (**A**) and ovarian (**B**) cancers. The red-to-blue color gradient on maps is scaled to indicate up- to down-regulation of gene expression values on a given map, respectively. The number of significantly deregulated genes at different FDR thresholds are given above each map. The largest numbers of de-regulated genes (*n* > 100 and *n* > 1000 at Fdr < 0.05) are indicated by * and **, respectively. See also Table 2.

The distribution of up- and down-regulated spot areas in the transcriptome portraits shows considerable variability between studied groups in both diseases. Upregulated genes in mutation carrier vs. non-carrier groups showed mirroring localizations in both diseases, indicating differential, partly antagonistic activation of expression programs. Additionally, common patterns were observed. For example, *BRCA1/2* mutations in breast cancer (BC) are uniquely associated with the upregulation of genes located in the right part of the map. For ovarian cancer (OV) one finds a similar result where upregulated genes in mutated samples were found in the lower right part. One also sees that somatic mutations of *BRCA1* or *BRCA2* associate with additional up-regulated spots compared to their germline counterparts (for example compare gBRCA1 and sBRCA1 portraits for breast cancer, or gBRCA2 vs. sBRCA2 portraits for ovarian cancer). To visualize these differences, we generated “difference portraits” between the groups. The analysis confirmed common gene expression deregulation in *BRCA1/2* mutation carrier patients compared to non-carriers both in the breast (Figure 2A) and ovarian cancers (Figure 2B). The strongest deregulation of gene expression is observed for the gBRCA1 group in breast cancer.

Top up- and down-regulated mutation-associated genes are presented in Table 2, as well as Supplementary Data 1 and 2. The results of differential gene expression analysis show that in BC and OV cancers, there is a variation of gene expression profiles in patients with somatic or germline *BRCA1*/*BRCA2* mutations, compared to patients without mutations, as well as between mutation types for the same gene. In the BC_gBRCA1 group, the highest expression was observed for *LINC02188*, *PROM1*, *ROPN1*, *GABRP*, and *FDCSP* genes. *LINC02188* has been shown to be upregulated in triple-negative breast cancers [22], while *PROM1*, *ROPN1*, *GABRP*, and *FDCSP* were previously associated with a cancer stem cell signature in a basal-like breast cancer phenotype [23,24]. 

In BC_sBRCA1 we observed overexpression of *S100A7* (psoriasin), a DNA damage-inducible gene associated with poor outcome in estrogen negative cancers [25], meanwhile indicating a good response to etoposide [26]. Breast cancer groups bearing germline and somatic *BRCA2* mutations as well as non-mutated patients were characterized by the upregulation of luminal subtype signatures such as *ESR1*, *TFF1*, *TFF2* [27]. In addition, the BC_gBRCA2 group showed overexpression of *RIMS4* indicative of estrogen-positive cancers [28] as well as *CNTFR*, which is shown to be deregulated in breast cancer [29], however, with unknown clinical impact. Finally, *ONECUT2* is upregulated in the BC_sBRCA2 group suggesting an association with cancer stem cell traits and expression of stemness-associated genes [30]. 

In the OV_*BRCA1* group, we observed overexpression of *RCCD1*, which was previously identified as a susceptibility locus for ovarian cancer [31], and of *SUSD2*, which promotes cancer metastasis and associates with cisplatin resistance [32]. OV_sBRCA1 samples were, in turn, characterized by the upregulation of *KRT16*, which is linked to migration, invasion, metastasis, and cancer stemness in ovarian cancer cells [33]. The OV_gBRCA2 group was associated with upregulated *OXTR*, which is essential for oxytocin-mediated inhibition of cell growth, invasion, and migration [34] by repressing the expressions of *MMP2* and *VEGF* [35]. Finally, OV_sBRCA2 was characterized by the up-regulation of zinc finger proteins (*ZNF613*, *ZNF329*, *ZNF530*, *ZNF347*), a gene family known to be involved in pathways of carcinogenesis, cancer progression, and metastasis formation [36,37]. 

Overall, more pronounced differential expression was observed in the case of breast cancer compared to ovarian cancer groups. Most activated genes associate with cancerogenic functions. Particularly, the core functionalities of differentially expressed genes in different groups often overlap and associate with cancer cell stemness, cancer progression, and metastasis development.

### 2.3. Alteration of Expression of BRCA1/2 and PARP (poly(ADP-ribose) polymerase) Genes between the Groups

**Figure 3:**
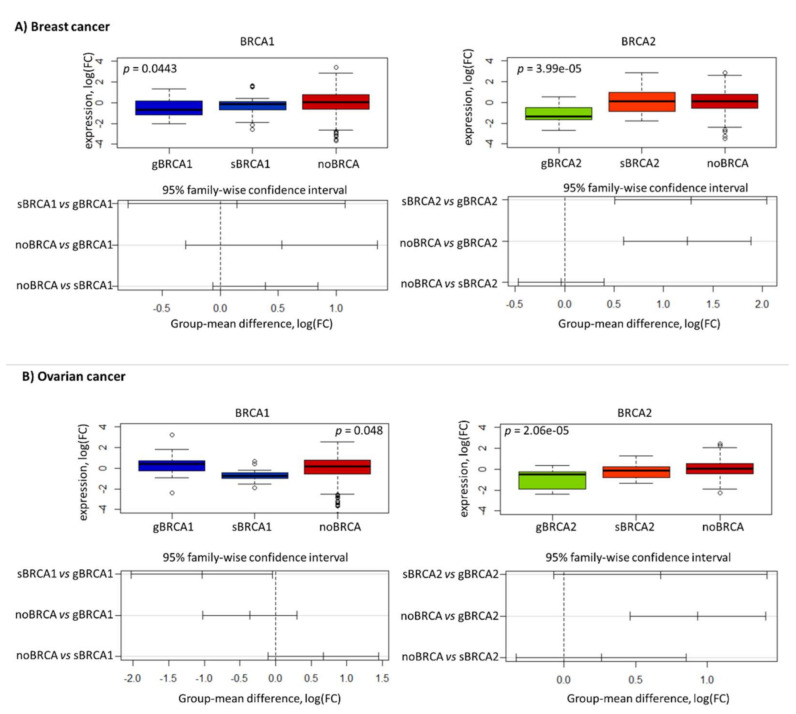
Mutation-associated *BRCA1* and *BRCA2* expression profiles in breast (**A**) and ovarian (**B**) cancers. gBRCA1 - germline *BRCA1* mutations, sBRCA1 - somatic *BRCA1* mutations, gBRCA2 - germline *BRCA2* mutations, sBRCA1 - somatic *BRCA1* mutations, nBRCA1/2 - no BRCA mutations. Significance was calculated using a one-way ANOVA test. Overall, results show the lowest expression levels of *BRCA1* observed in germline mutation carrier breast cancer groups and somatic *BRCA1* mutation carriers in ovarian cancers. Meanwhile, the lowest *BRCA2* expression is observed in germline mutation carriers both in breast and ovarian cancers, with an increase towards non-carriers.

Next, we compared the expression of *BRCA1* and *BRCA2* genes between the different groups. In breast cancer, their lowest expression was observed in patients bearing germline mutations followed by somatic mutation carriers and non-carriers (Figure 3A). On the contrary, in ovarian cancer, the expression of *BRCA1* was the lowest in samples with somatic *BRCA1* mutations compared to other groups, while the expression of *BRCA2* expression showed a similar pattern as in breast cancer (Figure 3B). These results show that mutation-associated decrease of *BRCA2* expression is consistently observed in both cancer types, while the expression of *BRCA1* varies in a cancer-specific fashion.

**Figure 4:**
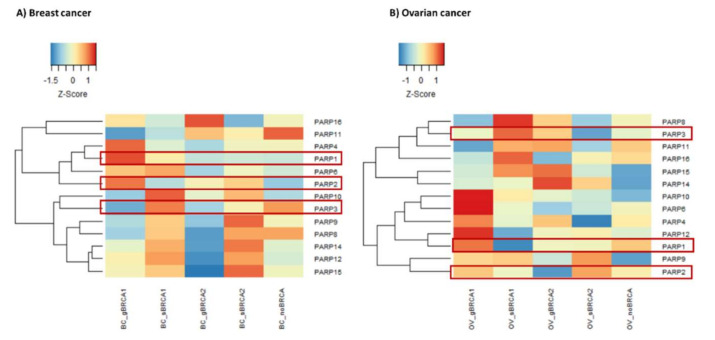
Heatmaps of poly(ADP-ribose) polymerase (*PARP)* family genes expression in breast (**A**) and ovarian cancer (**B**) subgroups stratified by *BRCA1* and *BRCA2* mutations. The results show that the highest expression of *PARP1* is associated with germline *BRCA1* mutations, while the expression of other PARP genes varies depending on the disease, gene, and mutation types.

In addition to *BRCA1* and *BRCA2*, we were also interested in evaluating the expression of poly(ADP-ribose) polymerase (*PARP*) genes, representing another key gene family in BC and OV pathophysiology and treatment [38,39,40]. *PARP* family genes have enzymatic and scaffolding activities and are implicated in DNA repair properties [41]. So, we performed a cluster analysis using expression values of 13 *PARP* genes available in our datasets (Figure 4). Of three *PARP* genes (PARP1–3) implicated in DNA repair [38], the highest expression of *PARP1* was observed in germline *BRCA1* carriers in both breast and ovarian cancers. Moreover, in any group, the expression of at least one *PARP1–3* gene was increased. Expression of other *PARP* genes was higher in somatic *BRCA1* and *BRCA2* carriers in breast cancer, and in gBRCA1, sBRCA1, and gBRCA2 in ovarian cancers. 

Taken together, these results indicate significant variability of transcriptomic programs in breast and ovarian cancers associated with germline and somatic mutations in *BRCA1* and *BRCA2* genes. Moreover, our results show that the patterns of expression of *BRCA* genes as well as *PARP* family genes vary in groups stratified by mutations as well as cancer types.

### 2.4. Functional Context of Gene Expression Deregulation Associated with BRCA Mutations

Differential gene expression analysis showed only subtle variation between germline and somatic mutations for *BRCA1* and *BRCA2* genes in ovarian and breast cancers. However, it is well known that even weak alterations of the expression of multiple genes can cause dramatic activity changes of biological pathways if they act in a concerted fashion [42,43,44]. To evaluate the functional context of such concerted changes in gene expression on the level of biological processes and pathways, we used Gene Set Z-score (GSZ) analysis [45].

**Figure 5:**
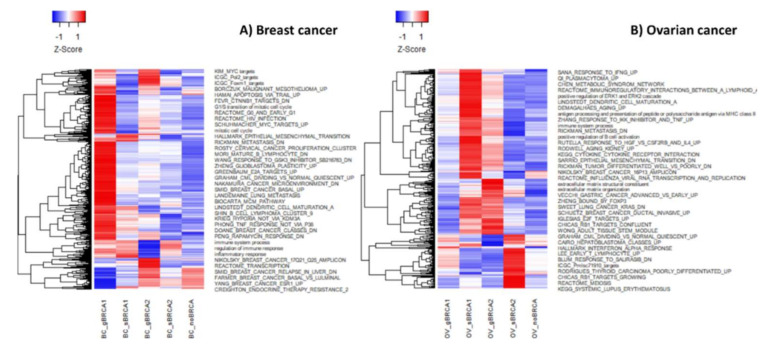
Clustering of deregulated *BRCA1*- and *BRCA2*-associated functional categories in the breast (**A**) and ovarian (**B**) cancers. One sees that the most prominent deregulations in breast cancer are linked to gBRCA1 in breast and sBRCA1/gBRCA2 in ovarian cancers, respectively. The gene sets upregulated in the gBRCA1 group include basal-phenotype signatures, immune/inflammatory response. In ovarian cancers, the upregulated functional categories are associated with immunity response (sBRCA1/gBRCA2) and chromosome/telomere maintenance (sBRCA2). More detailed clustering according to gene set types available in Supplementary Figures S1–S5.

In breast cancer, germline *BRCA1*, as well as somatic *BRCA1* and *BRCA2* mutations, were markedly associated with elevated immune system signatures, while cell proliferation/mitotic cell cycle and DNA repair were exclusively linked with germline mutations in *BRCA1*. On the other hand, germline *BRCA2* mutations showed upregulation in functional categories of protein transport and nucleosome assembly. Consistent with previous reports, germline and, to a lesser extent, somatic *BRCA1* mutations were associated with basal breast cancer phenotype-related functional gene sets, while germline and somatic *BRCA2* mutations were associated with luminal phenotype-related gene sets [46]. Simultaneously, all mutation-associated groups demonstrate upregulation in epithelial-mesenchymal transition (EMT)-related processes and suggest more aggressive and metastatic cancer subtypes compared with non-mutated breast cancer (Figure 5A). 

In contrast, in the ovarian cancer dataset, most of the deregulations in functional gene sets were associated with somatic *BRCA1* and germline *BRCA2* mutations (Figure 5B). *sBRCA1* and gBRCA2 mutation carriers were characterized by the upregulation of inflammatory/immune response, cytokine signaling, and T cell activation, as well as EMT and *KRAS* signaling. These stromal and inflammatory phenotypes were opposed by more proliferative ones in the case of sBRCA2 mutations, which is strongly associated with cell cycle, cell proliferation, and telomere maintenance functionalities.

**Figure 6:**
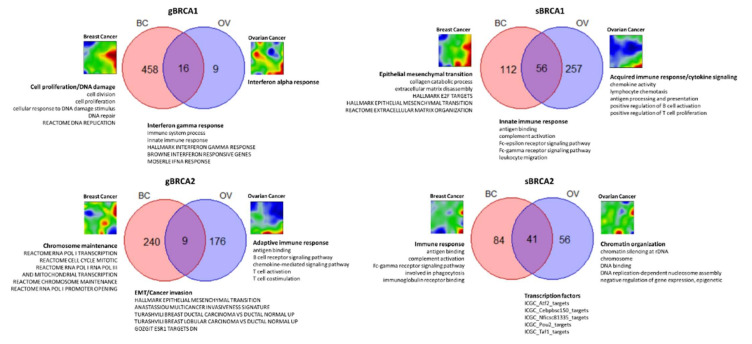
Overlap of functional gene sets upregulated in mutation associated group transcriptome landscapes in breast (BC) and ovarian (OV) cancers.

Next, we compared to what extent functional gene set deregulation associated with mutation type and mutated gene were shared across the breast and ovarian cancers (Figure 6). The results showed that there is a little overlap of upregulated functional gene sets associated with germline mutations both for *BRCA1* and *BRCA2* genes. In contrast, the upregulated gene sets considerably overlap in somatic mutation groups for both genes. In breast cancer, the germlines *BRCA1*, *BRCA2*, as well as somatic *BRCA1* were associated with DNA damage/repair, cell cycle, chromosome maintenance, and transcription. On the other hand, the same mutations in ovarian cancer were associated with adaptive and innate immunity as well as inflammatory gene sets. Interestingly, the functional associations were reversed in somatic *BRCA2* groups: In breast cancer, they were associated with immune response, while in ovarian cancer these mutations were associated with chromosome organization and maintenance.

Overall, our results indicate that the transcriptome landscape in breast and ovarian cancers are linked to a range of deregulated biological functions, mainly centered around DNA damage repair/cancer expansion as well as immune/inflammatory response. However, the context of the deregulation of functional processes largely depends on the disease, gene, and mutation types.

### 2.5. Phenotype and Survival Associations of BRCA1/2 Mutations in Cancers

**Figure 7:**
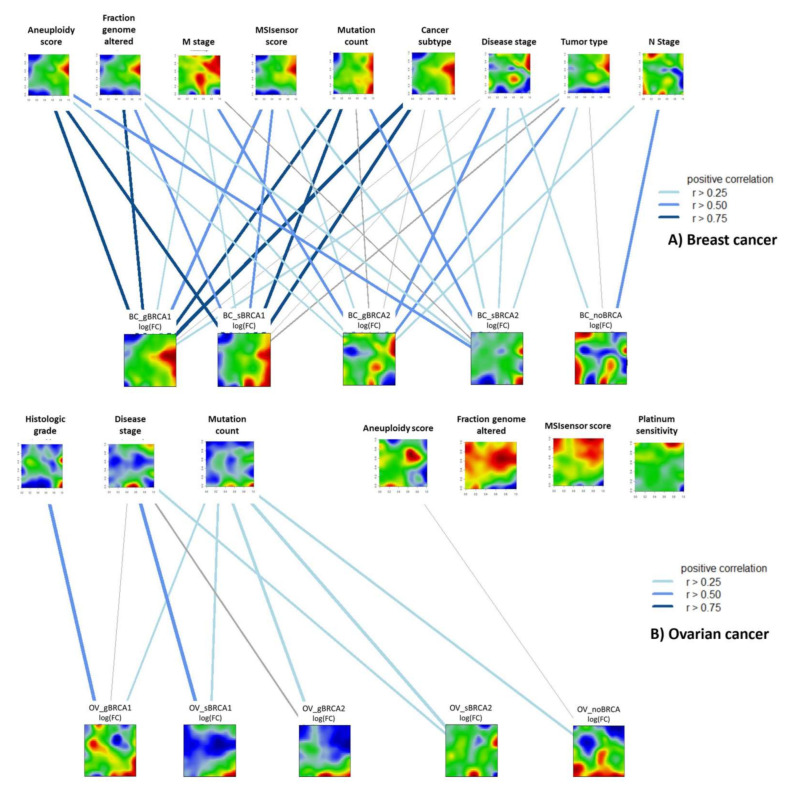
Phenotype-transcriptome associations in the breast (**A**) and ovarian (**B**) cancers. Phenotype maps were generated based on linear regression coefficients between corresponding characteristics and metagene expression profiles across all samples in a given dataset (See Materials and methods section). The similarity between phenotype and transcriptome was calculated for each studied group separately using Pearson’s correlation coefficient.

Next, we created phenotype maps based on regression coefficients between clinical data and transcriptome metagene profiles in studied groups. Phenotype portraits reflect the mutual association between deregulated gene clusters and the respective phenotype characteristics. The overlap of spot areas on phenotype and transcriptome maps indicate the mutual correlation between these parameters. For example, the upregulated gene spot on the right part of the BC_gBRCA1 portrait well overlaps with the corresponding spot on the aneuploidy score portrait (Figure 7A), indicating the positive correlation between gene expression and aneuploidy score.

In breast cancers, mutation-associated transcriptome portraits were associated with increased aneuploidy, the fraction of genome alterations, and microsatellite instability, consistent with previous results demonstrating the causal link between mutations in DNA damage response genes genome instability [47,48]. Meanwhile, both germline and somatic *BRCA1* mutations were strongly correlated with basal cancer subtype. The strongest association between transcriptome portraits and advanced neoplasm stage, as well as T (size and extent of the main tumor) and M (degree of metastasis) stages, were observed for gBRCA2. Furthermore, the non-carrier group showed the strongest association with N stage (regional lymph node infiltration) (Figure 7A), which is a negative prognostic factor in non-carrier breast cancer patients [49]. 

Ovarian cancers associate with advanced histologic grade and disease stage (*BRCA1* and *BRCA2* mutation groups, respectively), similarly to the results reported by Lakhani et al. (2004) [50]. No positive association was observed for aneuploidy score, microsatellite instability, overall genome alterations, and platinum sensitivity in any of the groups (Figure 7B) consistent with previous reports [51]. Overall, our results suggest that in breast cancers, mutations in BRCA genes stronger associate with phenotypes, while ovarian cancers are characterized by higher heterogeneity.

The analysis of the association of treatment regimens with transcriptome profiles indicated that the patients bearing different types of mutations in *BRCA1* and *BRCA2* genes have received different treatments (Supplementary Figure S6A,B). The prescribed drugs partially overlapped between breast and ovarian cancer patients; however, no consistency in gene/mutation and drug maps has been observed in studied datasets. For example, the paclitaxel map correlated BC_gBRCA1 and BC_sBRCA1 transcriptome portraits, while in the ovarian cancer dataset, it correlated with OV_sBRCA1 and OV_noBRCA. 

**Figure 8:**
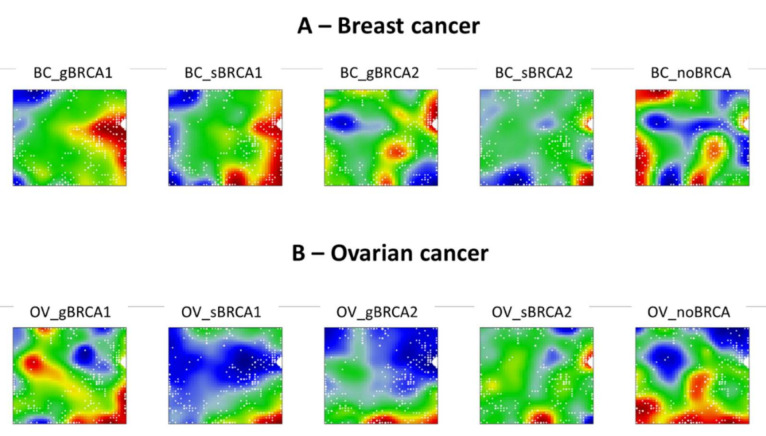
Olaparib gene signature distribution on the transcriptome landscapes in the breast (**A**) and ovarian (**B**) cancers. White dots represent Olaparib-related genes mapped to the corresponding metagenes on a given transcriptome landscape. It can be noted that signature genes are predominantly mapped on or around upregulated (red) spots on each portrait. This suggests different action effects of Olaparib depending on the disease, mutation type, and gene affected.

Finally, we were interested in how PARP inhibitor treatment-related genes are mapped on transcriptome portraits of studied groups. We populated PARP inhibitor gene signatures from previously published articles [52,53,54] and projected them as white color dots onto transcriptome maps. The results showed that the majority of PARP inhibitor signature genes were located in or near the deregulated gene clusters across the transcriptome landscapes of studied disease groups, implying that signature genes (most affected by the drug or indicative for drug efficacy) are among the most upregulated ones in the respective groups. It has also become apparent that different PARP inhibitor-associated genes map to different deregulated spots depending on the disease, mutated *BRCA1* or *BRCA2* gene, and mutation type (Figure 8A,B).

**Figure 9:**
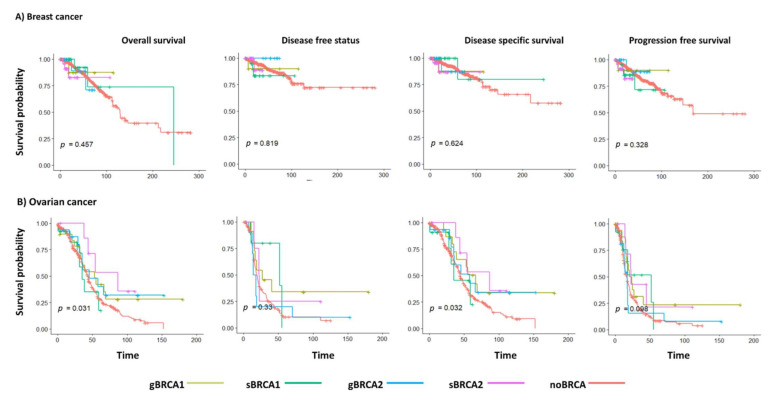
Survival associated with *BRCA1* and *BRCA2* mutations in the breast (**A**) and ovarian cancers (**B**). Significance was calculated using a Cox proportional hazards regression model.

We have also analyzed the survival data of BC and OV associated with BRCA mutations. In breast cancer, no differences were observed in overall, disease-free, disease-specific, and progression-free survival in studied groups (Figure 9A). In contrast, overall survival, as well as disease-specific survival in OV, were slightly better in gBRCA1 and sBRCA2 groups, respectively (Figure 9B). 

## 3. Discussion

The prevalence of germline and somatic *BRCA1*/2 mutations are highest in breast and ovarian cancers and their presence is associated with an indication for PARP-inhibitor therapy. However, there are growing reports indicating differences in clinical outcomes, chemotherapy sensitivity, as well as variability of BRCA gene expression depending on the mutation types, at least in ovarian cancers [55,56]. Less clear information is available for breast cancers [57,58]. Moreover, there are virtually no studies aimed at understanding the influence of mutation types on the molecular mechanisms associated with breast and ovarian cancers. Usually, germline mutation phenotypes are being compared with the sporadic cases, however, without specifically focusing on the somatic mutations affecting the same gene [19,21,59].

In this study, we evaluated perturbations in transcriptome landscapes as a function of the disease (breast vs. ovarian cancers), the gene (*BRCA1* vs. *BRCA2*), and the mutation type (somatic vs. germline) by applying a multilayer self-organizing maps approach on the next generation RNA-sequencing data from TCGA-OC and TCGA-BRCA projects. Our results clearly showed the “multivariate” character of these perturbations. 

The most notable changes in our study refer to the mutation types in breast and ovarian cancers. So far, the differential effects of germline and somatic mutations in *BRCA1* or *BRCA2* genes on the transcriptome and associated functional processes have not been studied to a great extent as opposed to the clinical effects. A recent transcriptomic study reported similarity between germline and somatic mutations of *BRCA1*/2 genes in breast cancer [60] in agreement with subtle differences in gene expression observed in this study (Figure 1). However, our functional analysis revealed a series of novel details. In ovarian cancers, we find an enhanced immune signature in somatic *BRCA1* and germline *BRCA2* carriers in agreement with previous reports [61], in which, however, differentiation between mutation types has not been explored. The most profound differences in ovarian cancers were observed between the transcriptomes of germline and somatic *BRCA2* mutated cases. While the former upregulated functions were related to the immune response, the latter was associated with chromatin silencing, telomere organization, and cell cycle checkpoints. On the other side, both, germline *BRCA1* and *BRCA2* mutations in breast cancer were associated with the latter functions, namely DNA damage, proliferation and chromosomal organization, and telomere maintenance, while somatic mutations in those genes were mostly linked to the immune and inflammatory response. Indeed, it has been previously shown that germline *BRCA1*/2 cancers are less immunologically active, which could be attributed to the compromised immune system because of the mutations [62]. In addition, there is evidence that germline and somatic mutations may have different effects on the structure and function of the encoded protein as well as be linked to different classes of diseases [63]. Finally, we also observed differential expression of *BRCA1*, *BRCA2*, and *PARP* family genes depending on the mutation type, the gene, and the disease. These observations are of special importance since *PARP* inhibitors are thought to be equally effective in treating *BRCA1-* or *BRCA2*-linked ovarian or breast cancers [64].

Recent studies indicate that the function of PARP genes extends beyond maintaining genome stability and is gene-specific. For example, PARP-1, but not PARP-2, is involved in the formation of immunosuppressive macrophage phenotypes in the tumor microenvironment after olaparib treatment and further modulate immunosuppression by enhancing PD-1 expression [65]. Furthermore, PARP-2 is essential for thymocyte development, while PARP-1 regulated Treg development [66]. Even less is known about the biological functions of other *PARP* genes in the context of regulation of tumor microenvironment and other extra functions. Collectively, this and other data suggest that targeted therapies with PARP inhibitors should consider the intended action on aberrant pathways (e.g., directly activated/deactivated by mutations as discussed), but also the accompanying effects such as modulation of the tumor microenvironment (as indicated by changed “inflammatory signatures”), and provide indications for research into combinations of cytotoxic and immunotherapies to increase treatment efficacy.

Besides differential transcriptome response to germline and somatic mutations, we also observed disparity in deregulation of functional modules associated with the *BRCA1* and *BRCA2* genes. Both *BRCA1* and *BRCA2* are crucial in maintaining genomic stability through double-strand DNA repair by homologous recombination [1,2]. However, both being tumor suppressors, *BRCA1* and *BRCA2* seem to be involved in different stages of DNA damage response and repair [67,68]. Moreover, *BRCA1* mutations are usually associated with estrogen receptor deficiency, which is not the case of *BRCA2* mutations [69]. Furthermore, *BRCA1* serves as a co-transcription factor for OCT-1, c-Myc, ERα, p53, Smad3, and others [70]. Our results also indicate that there are considerable differences in deregulation for gene sets either associated with *BRCA1* or *BRCA2* genes. Thus, our results agree with previous reports on distinctive mechanisms associated with the dysfunction of *BRCA1* or *BRCA2* in cancers [19,62]. 

Finally, we observed considerable differences in the deregulation of transcriptome for the same mutation when comparing breast and ovarian cancers. Consistent with previous reports BRCA mutations in breast cancer were mostly associated with regulation of cell cycle, DNA damage, and cell proliferation in breast cancer [71], and with immune system-related processes in ovarian cancer [61], which may be an indicator of differential role of *BRCA1* and *BRCA2* in the pathogenesis of these diseases. Previous studies have already suggested mechanisms of how DNA damage may trigger immune response [72,73]; however, the question of why its intensity is higher in ovarian rather than in breast cancer remains open. 

The principal limitation of our study is linked to the sample size. Even in large datasets such as TCGA-OV and TCGA-BRCA, there are still a small number of mutation carriers for *BRCA1* and *BRCA2* genes, especially when they are stratified into groups by the mutation type. This was also a reason for preventing further stratification of biallelic and monoallelic mutations. However, a small sample size is compensated to a certain degree by the exploitation of the multi-layer SOM approach. The SOM-based training ensures that the obtained metagene clusters incorporate the whole variety of the expression profiles existing in the high-dimensional data [74,75]. As a result, we were able to take advantage of various features of the TCGA-OV and TCGA-BRCA datasets regardless of the uneven distribution of the features among the sample groups. Larger samples of mutation carriers will enable a more accurate exploration of the expression profiles and metagene-linked molecular mechanisms associated with the different types of BRCA mutations in breast and ovarian cancers in future investigations.

## 4. Materials and Methods

### 4.1. Data Sources and Preprocessing

RNA-seq data for breast (BC, TCGA-BRCA project) and ovarian (OV, TCGA-OV project) cancer samples were obtained from The Cancer Genome Atlas Program (TCGA) repository [76]. OV dataset contains RNA-seq gene expression profiles from 338 cases with serous cystadenocarcinoma; BC dataset contains RNA-seq data from 694 cases. Only RNA-seq data from primary tumor samples from the first vial (“-01A”, refer to the TCGA barcode page) were selected, to ensure that the earliest time point samples were included in further analyses. 

Raw RNA-seq counts were filtered to remove transcripts with zero 0 counts across all samples, then were log10 transformed, and centralized against global mean expression values.

Mutation status of *BRCA1* and *BRCA2* were obtained from the cBio genomics portal [76], which contains four TCGA-BRCA and three TCGA-OV partially overlapping datasets. Information about the germline and somatic status of *BRCA1* and *BRCA2* mutations was obtained from accompanying publications ([46] and [77] for BC and OV, respectively) as well as from GDAC Firehose data (https://gdac.broadinstitute.org/). As samples without BRCA mutations, we selected those samples that did not have reported BRCA mutations in any of the mentioned cBio portal datasets.

Clinical, survival, and treatment-related information was obtained from the TCGA and cBio data portals.

### 4.2. Transcriptome Portrayal with Self-Organizing Maps

Transcriptome analysis was performed using a multi-layer self-organizing maps (SOM) machine learning approach described in detail previously [74,75,78,79]. In the present study, we performed two-layer training, each containing the transcriptomic dataset from one TCGA project. The SOM approach represents dimension reduction that translates M = 32,039 gene expression profiles in each layer into K = 2025 (45 × 45) metagenes, each representing a cluster of genes with similar profiles of expression across samples. The SOM training algorithm distributes the *N* genes over the K metagenes using the minimal Euclidean distance of the expression profiles within and between layers as a similarity measure. It clusters genes with similar profiles in the same or closely located metagenes. Each metagene profile can be interpreted as the mean profile averaged over all gene profiles referring to the respective metagene cluster. The metagene expression values of each sample are visualized (expression portrayal) by arranging them into a two-dimensional 45 × 45 grid and by using maroon to blue colors for maximum to minimum expression values in each of the portraits. Multi-layer SOM ensures that each of the layers is projected into identical SOM-space formed of metagenes that contain the same single genes at the same position of the metagene-grid in each of the layers, which made them directly comparable across the layers [79].

### 4.3. Gene Set Z-Score Analysis

Functional analysis of co-regulated genes in spot modules was performed using Fisher Exact test and Gene Set Z-score algorithm [46] based on gene set collection available in oposSOM package [78], which includes gene sets obtained from GSEA-repository, “hallmarks of cancer”, NIH Roadmap Epigenomics Consortium for chromatin state-related gene sets, as well as from the retrieved from various publications.

### 4.4. Phenotype Mapping to Co-Expressed Gene Modules

Phenotype information such as medication, disease stage, and grade was obtained from the TCGA data portal. Phenotype maps were constructed based on coefficients of logistic (categorical variables) or linear regression (numerical variables) between phenotype categories and metagene expression. Correlation between phenotype and transcriptomic maps was assessed using Pearson’s correlation coefficient.

### 4.5. Survival Analysis

Survival analysis (overall survival, disease-free survival, disease-specific survival, and progression-free survival) depending on the status of *BRCA1*/2 gene mutations was performed using the Cox proportional hazards regression using ***survival*** and ***survminer*** R packages.

### 4.6. Data Availability

The complete analysis results were deposited as supplementary datasets in the open-access repository Zenodo (https://zenodo.org/) [80].

## 5. Conclusions

Transcriptomic landscapes of breast and ovarian cancers show considerable variation depending on the affected gene (*BRCA1* or *BRCA2*) as well as the mutation type (somatic or germline). Our results warrant further investigations with larger groups of mutation carriers that could pave a way for a better understanding of the fine molecular mechanisms of breast and ovarian cancers.

## Figures and Tables

**Table 1:** Sample groups in the breast (TCGA-BRCA) and ovarian (TCGA-OV) cancer datasets were used as separate layers in self-organizing maps (SOM) analysis.

	TCGA-OV RNA-Seq Dataset *n* (%)	TCGA-BRCA RNA-Seq Dataset *n* (%)
Germline *BRCA1*	19 (5.6%)	12 (1.7%)
Somatic *BRCA1*	14 (4.1%)	28 (4.0%)
Germline *BRCA2*	16 (4.7%)	15 (2.2%)
Somatic *BRCA2*	8 (2.4%)	26 (3.7%)
No *BRCA* mutations	281 (83.1%)	613 (88.3%)

**Table 2:** Groupwise analysis of differential expression analysis in breast and ovarian cancers.

Groups	Brest Cancer ID	Symbol	logFC	Fdr	Ovarian Cancer ID	Symbol	logFC	Fdr
gBRCA1 vs. gBRCA2	ENSG00000135069 ENSG00000102854 ENSG00000162989 ENSG00000173467	FDCSP PSAT1 KCNJ3 AGR3	5.12 3.86 5.03 5.16	0.06 0.04 * 0.05 * 0.04 *	ENSG00000204934 ENSG00000166965	ATP6V0E2-AS1 RCCD1	1.11 1.01	0.12 0.2
gBRCA1 vs. noBRCA	ENSG00000181617 ENSG00000094755 ENSG00000160182 ENSG00000091831	FDCSP GABRP TFF1 ESR1	4.58 4.57 −4.35 −4.58	0.01 ** 0 ** 0 ** 0 **	ENSG00000099994 ENSG00000076344 ENSG00000267327 ENSG00000259129	SUSD2 RGS11 LINC00648	1.37 1.3 −1.32 −1.67	0.23 0.23 0.06 0.11
gBRCA1 vs. sBRCA1	ENSG00000261175 ENSG00000171243 ENSG00000091831 ENSG00000101210	LINC02188 SOSTDC1 ESR1 EEF1A2	3.3 3.06 −2.94 −3.3	0.14 0.15 0.19 0.13	-	-	-	-
gBRCA1 vs. sBRCA2	ENSG00000094755 ENSG00000261175 ENSG00000160180 ENSG00000091831	GABRP LINC02188 TFF3 ESR1	4.77 4.23 −3.72 −4.11	0.01 ** 0.01 ** 0.03 * 0.02 *	ENSG00000149527 ENSG00000275426 ENSG00000256087 ENSG00000176024	PLCH2 ZNF432 ZNF613	1.53 1.07 −0.81 −0.87	0.19 0.19 0.19 0.2
gBRCA2 vs. noBRCA	ENSG00000122756 ENSG00000176406 ENSG00000237940 ENSG00000159763	CNTFR RIMS2 LINC01238 PIP	2.16 1.96 −1.94 −2.41	0.09 0.13 0.13 0.22	ENSG00000180914 ENSG00000099953 ENSG00000100473 ENSG00000255571	OXTR MMP11 COCH MIR9-3HG	1.84 1.81 −1.1 −1.64	0.07 0.12 0.23 0.12
gBRCA2 vs. sBRCA2	ENSG00000122756 ENSG00000135097 ENSG00000139618 ENSG00000196092	CNTFR MSI1 BRCA2 PAX5	2.36 2.22 −1.37 −1.74	0.24 0.21 0.21 0.2	ENSG00000180914 ENSG00000101445 ENSG00000147536 ENSG00000123219	OXTR PPP1R16B GINS4 CENPK	3.24 2.4 −1.02 −1.15	0.11 0.15 0.2 0.2
sBRCA1 vs. gBRCA2	ENSG00000159184 ENSG00000188257 ENSG00000101098 ENSG00000263639	HOXB13 PLA2G2A RIMS4 MSMB	2.81 2.49 −3.2 −3.65	0.12 0.1 0.12 0.1	-	-	-	-
sBRCA1 vs. noBRCA	ENSG00000178372 ENSG00000186832 ENSG00000256612 ENSG00000153002	CALML5 KRT16 CYP2B7P CPB1	2.11 1.82 −2.27 −2.54	0.01 ** 0.05 * 0.03 * 0 **	ENSG00000186832 ENSG00000200087 ENSG00000072041 ENSG00000130294	KRT16 SNORA73B SLC6A15 KIF1A	1.93 1.64 −1.87 −2.39	0.17 0.18 0.13 0.16
sBRCA1 vs. sBRCA2	-	-	-	-	ENSG00000155966 ENSG00000246695 ENSG00000012048 ENSG00000180071	AFF2 RASSF8-AS1 BRCA1 ANKRD18A	2.24 1.72 −1.75 −1.91	0.18 0.22 0.17 0.09
sBRCA2 vs. noBRCA	ENSG00000119547 ENSG00000089692 ENSG00000213759 ENSG00000082175	ONECUT2 LAG3 UGT2B11 PGR	1.34 1.04 −1.77 −2.13	0.24 0.23 0.12 0.1	ENSG00000196787 ENSG00000204860 ENSG00000259439 ENSG00000187908	HIST1H2AG FAM201A LINC01833 DMBT1	1.89 1.46 −1.85 −1.92	0.15 0 ** 0.12 0.04 *

*-fdr ≤ 0.05, **-fdr ≤ 0.01.

## Data Availability

The complete analysis results are deposited as supplementary datasets in the open-access repository Zenodo (https://zenodo.org/record/4326452) as well as supplementary materials of this article.

## Supplementary Materials

The following are available online at https://www.mdpi.com/1422-0067/22/3/1266/s1, Supplementary Data 1: Pairwise differential expression of genes across groups in breast cancer, Supplementary Data 2: Pairwise differential expression of genes across groups in ovarian cancer, Figure S1: Clustering of deregulated BRCA1- and BRCA2-associated GO Biological Process functional categories in the breast (A) and ovarian (B) cancers, Figure S2: Clustering of deregulated BRCA1- and BRCA2-associated GO Molecular Function functional categories in the breast (A) and ovarian (B) cancers, Figure S3: Clustering of deregulated BRCA1- and BRCA2-associated transcription factor targets in the breast (A) and ovarian (B) cancers, Figure S4: Clustering of deregulated BRCA1- and BRCA2-associated “cancer hallmarks” in the breast (A) and ovarian (B) cancers, Figure S5: Clustering of deregulated BRCA1- and BRCA2-associated GSEA C2 functional categories in the breast (A) and ovarian (B) cancers, Figure S6: Drug transcriptome map relations for breast(A) and ovarian(B) cancers.
