# Supplementary material for: Transcriptome Patterns of BRCA1- and BRCA2- Mutated Breast and Ovarian Cancers
Source: Int J Mol Sci. 2021 Jan 28;22(3):1266. doi: 10.3390/ijms22031266 (PMC7865215; doi:10.3390/ijms22031266)
Supplement: Supplementary file 1 [file ijms-22-01266-s001.zip › SupplementaryFigures.pdf]

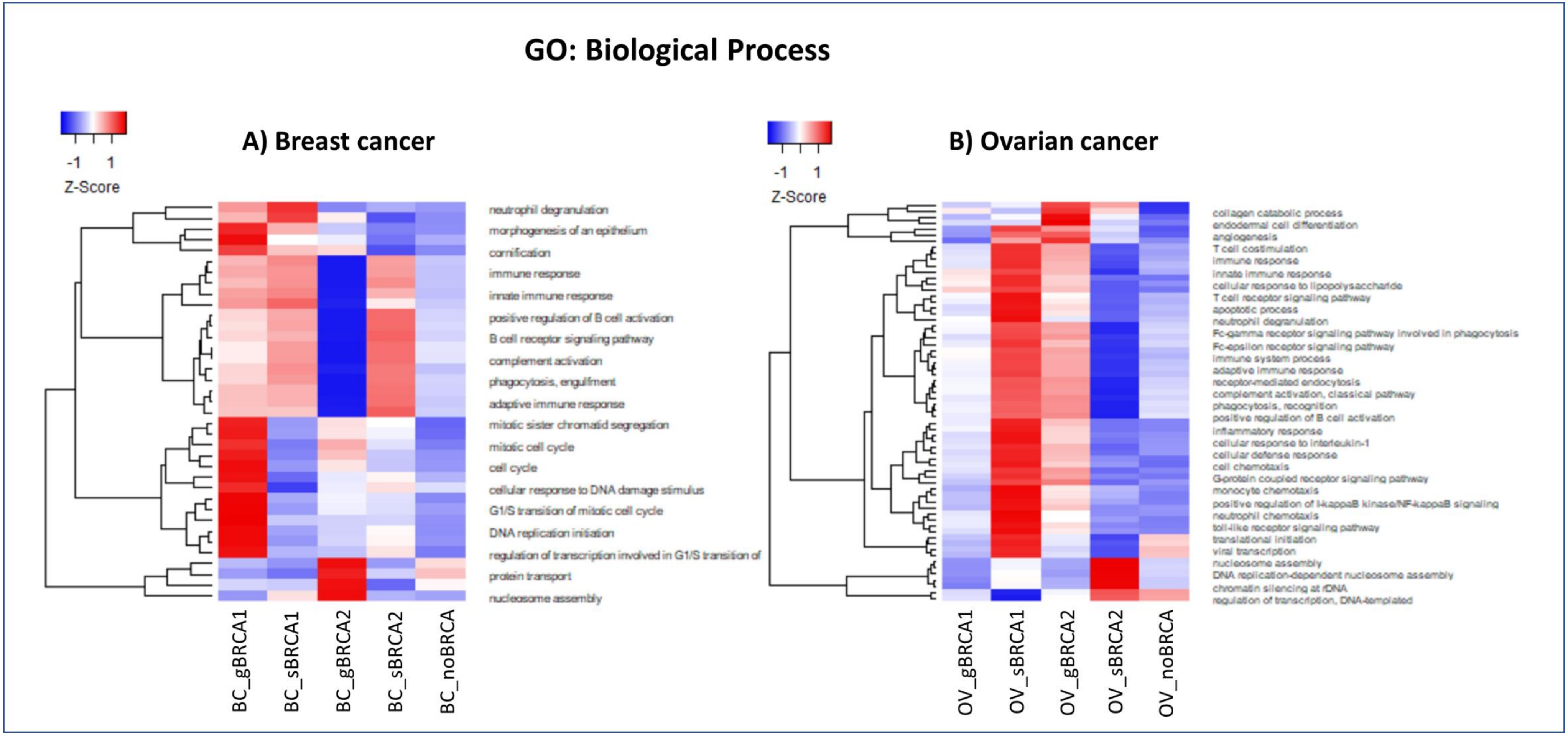

Figure S1. Clustering of deregulated BRCA1- and BRCA2-associated GO Biological Process functional categories in the breast (A) and ovarian (B) cancers.

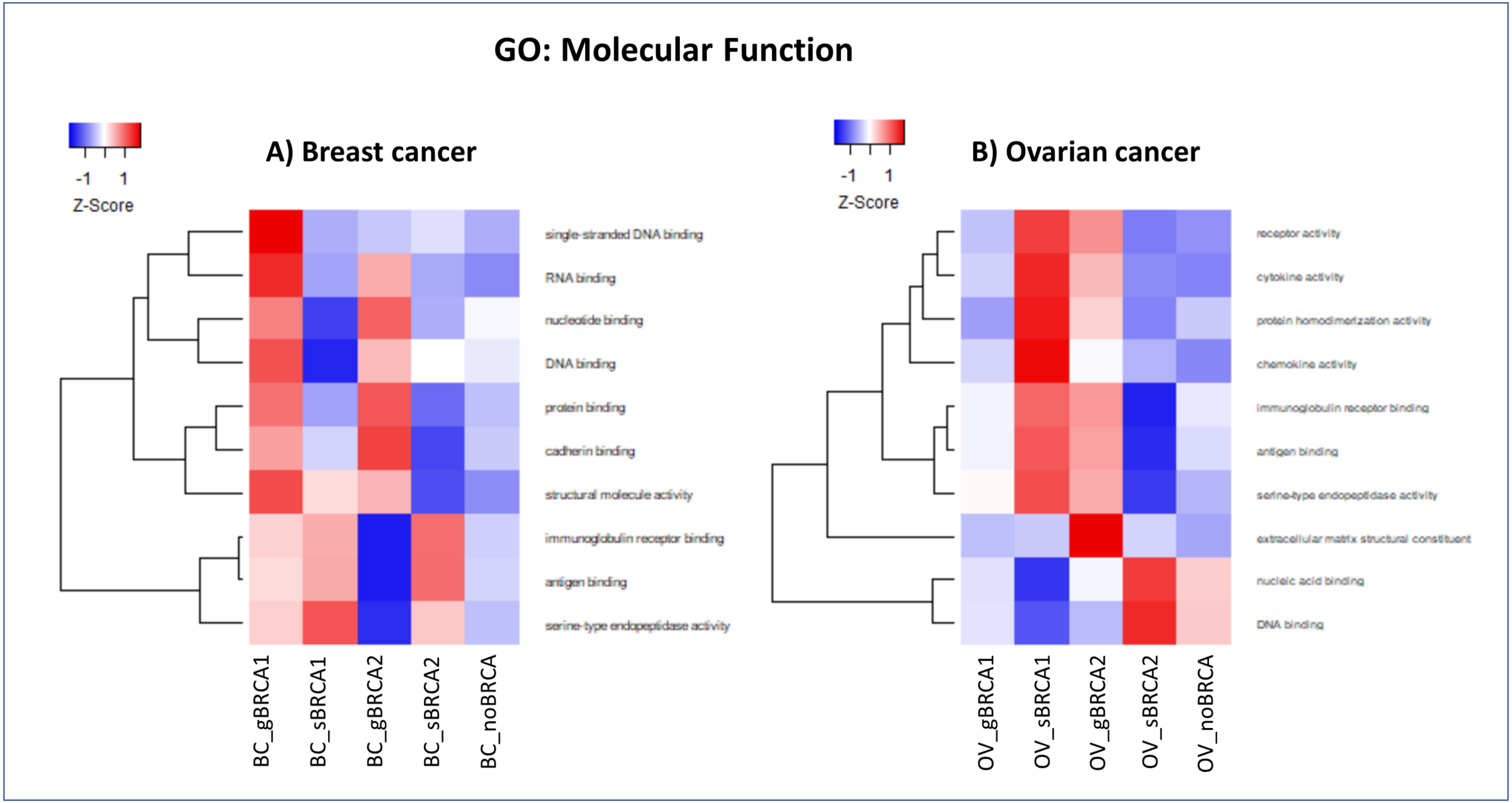

Figure S2. Clustering of deregulated BRCA1- and BRCA2-associated GO Molecular Function functional categories in the breast (A) and ovarian (B) cancers.

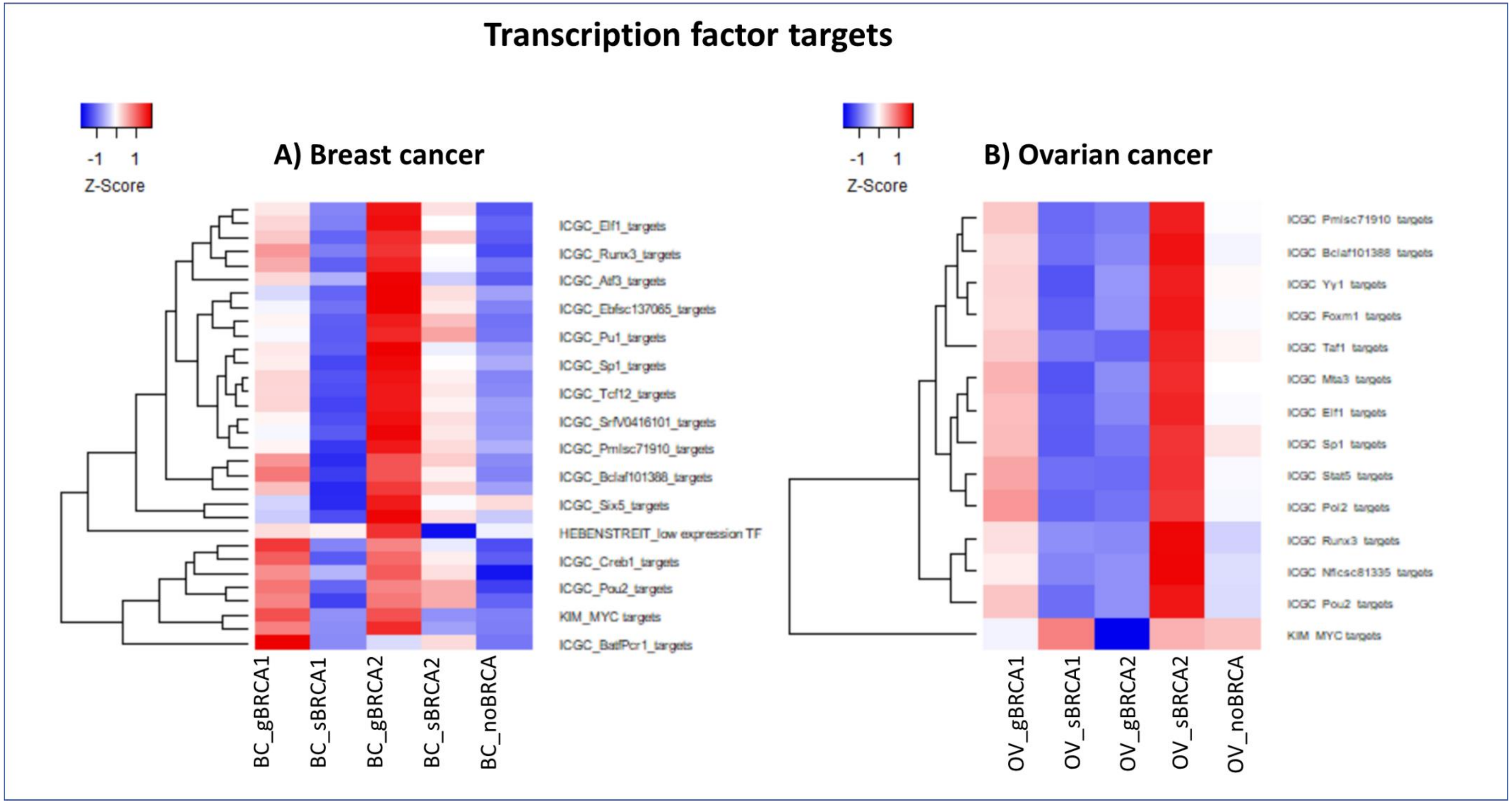

Figure S3. Clustering of deregulated BRCA1- and BRCA2-associated transcription factor targets in the breast (A) and ovarian (B) cancers.

Cancer hallmarks

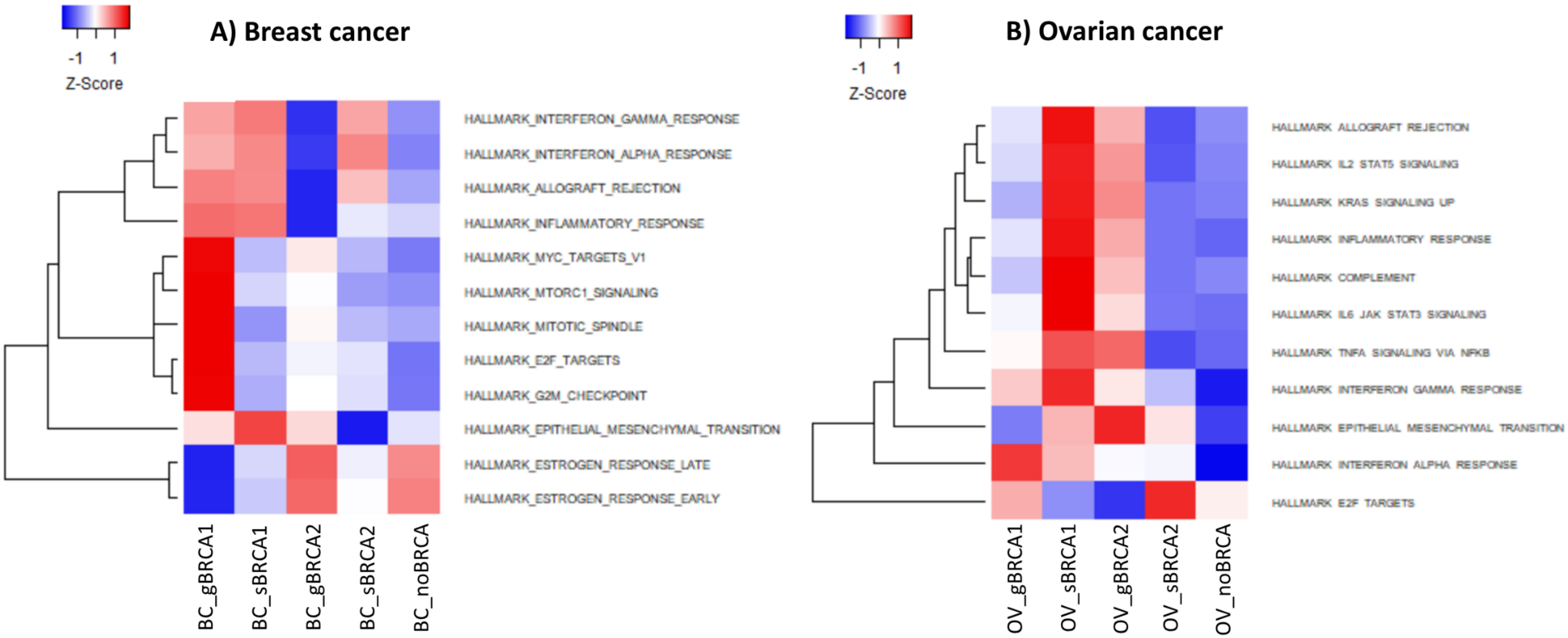

Figure S4. Clustering of deregulated BRCA1- and BRCA2-associated "cancer hallmarks" in the breast (A) and ovarian (B) cancers.

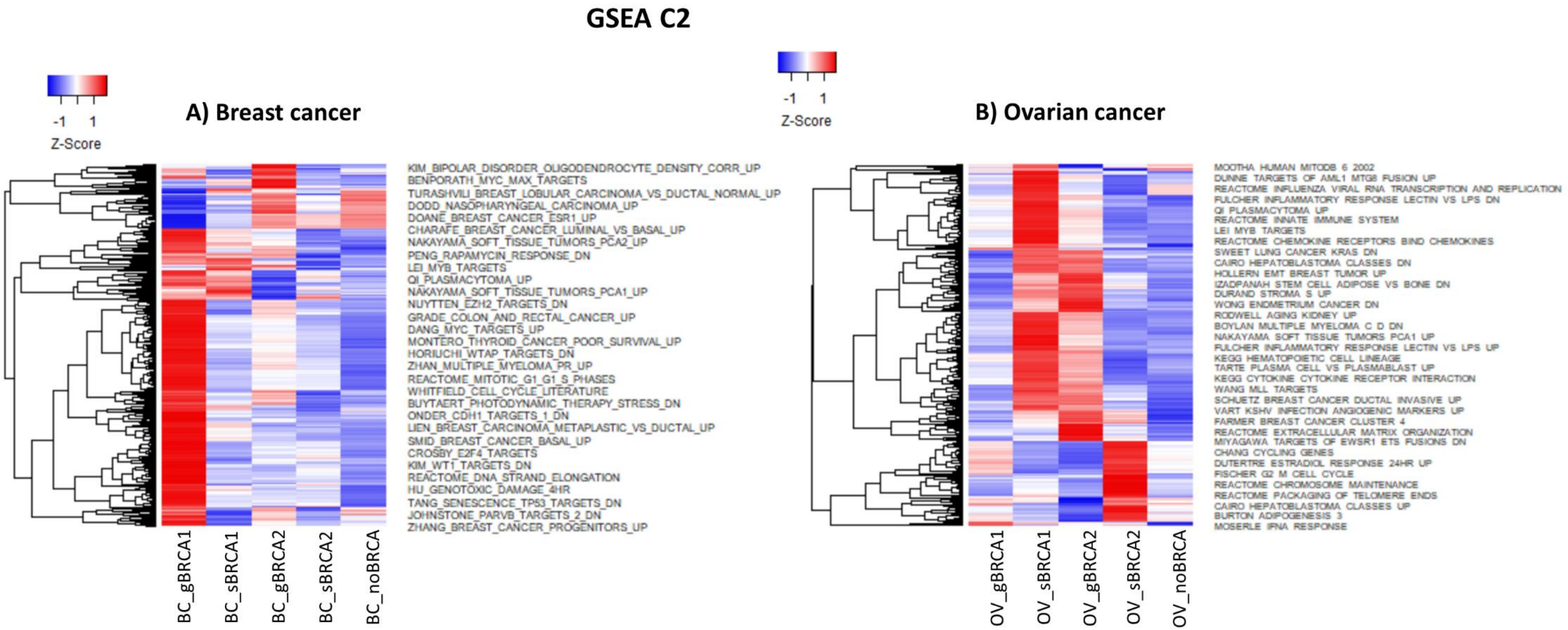

Figure S5. Clustering of deregulated BRCA1- and BRCA2-associated GSEA C2 functional categories in the breast (A) and ovarian (B) cancers.

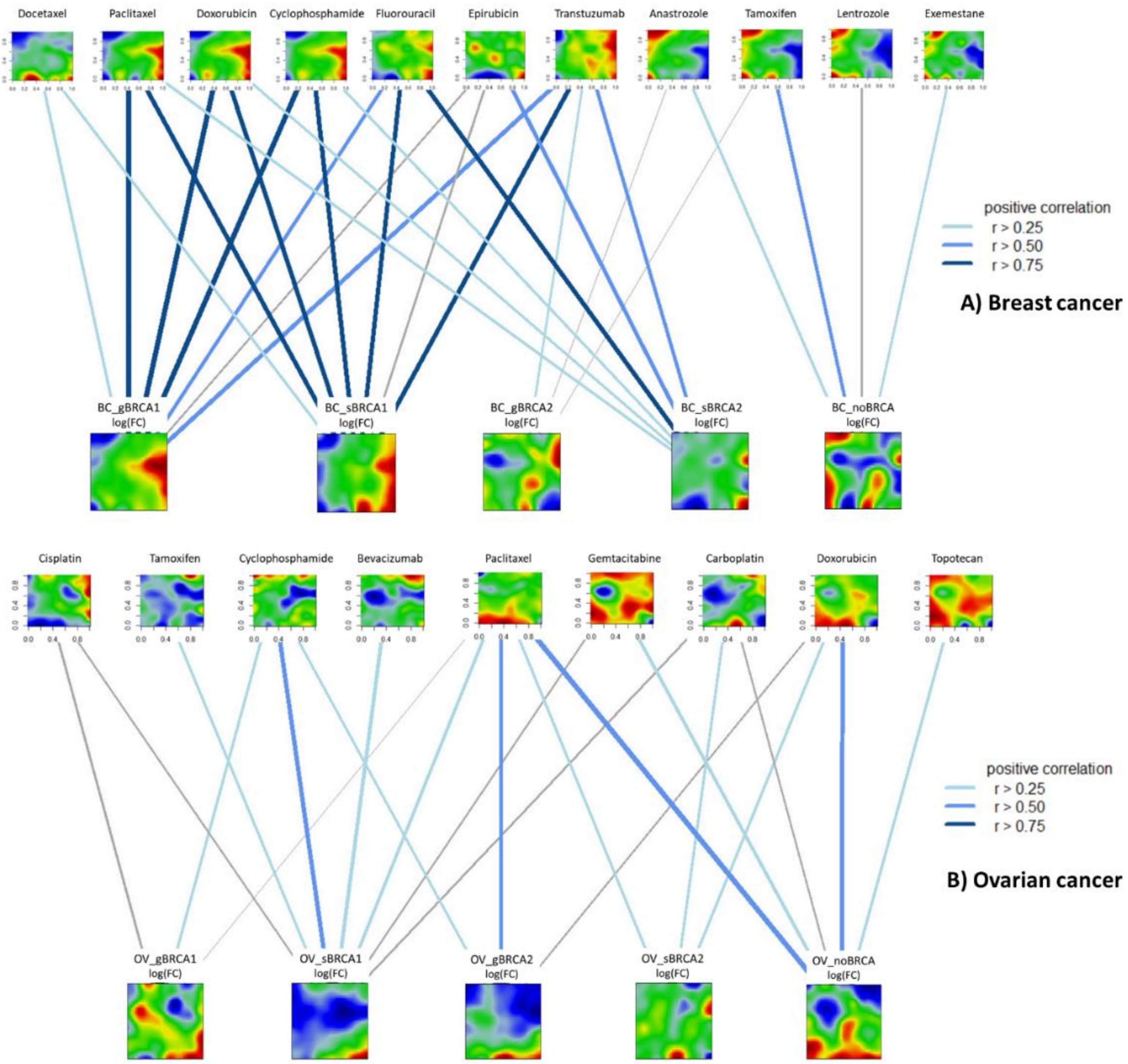

Figure S6. Drug transcriptome map relations for breast(A) and ovarian(B) cancers. Phenotype maps were generated based on linear regression coefficients between corresponding characteristics and metagene expression profiles across all samples in a given dataset (See Materials and methods section). The similarity between phenotype and transcriptome was calculated for each studied group separately using Pearson's correlation coefficient.
